# Supplementary material for: Path‐seq identifies an essential mycolate remodeling program for mycobacterial host adaptation
Source: Mol Syst Biol. 2019 Mar 4;15(3):e8584. doi: 10.15252/msb.20188584 (PMC6398593; doi:10.15252/msb.20188584)
Supplement: Supplementary file 1 — Appendix [file MSB-15-e8584-s001.pdf]

Appendix

Path-seq identifies an essential mycolate remodeling program for mycobacterial adaptation in host cells

Eliza J.R. Peterson, Rebeca Bailo, Alissa C. Rothchild, Mario L. Arrieta-Ortiz, Amardeep Kaur, Min Pan, Dat Mai, Abrar A. Abidi, Charlotte Cooper, Alan Aderem, Apoorva Bhatt, Nitin S. Baliga

Correspondence to: a.bhatt@bham.ac.uk and nitin.baliga@systemsbiology.org

List of Figures

Figure S1. Schematic of *in vivo* infection and isolation of alveolar macrophages from MTB infected mice……………………………………………………………………..…..……1

Figure S2. Schematic of *in vitro* infection…………………………………………….………2

Figure S3. Analysis of biological Path-seq replicates from *in vitro* infection…………..……..…3

Figure S4. Analysis of significantly differentially expressed genes from both *in vitro* and *in vivo* infection models………………………………………..……………………………......4

Figure S5. Consensus motifs from ChIP-seq peaks of *Rv0472c* overexpressed in MTB and *MSMEG_0916* overexpressed in MSM………..…………………………………………..…...5

Figure S6. Cell viability of Rv0472c and MSMEG_0916, overexpression strains…...……..……6

Figure S7. Growth of MSM overexpressing *MSMEG_0916* in 7H9 broth…….………………..…7

Figure S8. Assay of total mycolates in *MSMEG_0916* overexpression strain……...…………....8

Figure S9. Densitometric analysis of FAMES and MAMES in BCG *Rv0472c* overexpression strain………………………...………………………………………………………….......9

Figure S10. A 2D TLC analysis of FAMEs and MAMEs from BCG overexpressing *Rv0472*c..10

List of Tables

Table S1. Summary analysis from Path-seq data of alveolar macrophages (AMs) from MTB infected mice and extracellular MTB grown in 7H9 broth…………..…………………………11

Table S2. Comparison of significantly differentially expressed genes (DEGs) from in vitro Path-seq data with published microarray-collected transcriptomics data of MTB-infected macrophages………………………………..…………………………………………12

List of Expanded View Datasets (separate files)

Dataset EV1. Significantly differentially expressed genes of MTB from *in vivo* infection vs extracellular MTB using Path-seq.

Dataset EV2. Significantly differentially expressed genes of MTB from *in vitro* infection vs extracellular MTB using Path-seq.

Dataset EV3. Comparison of significantly differentially expressed genes between *in vivo* and *in vitro* infection.

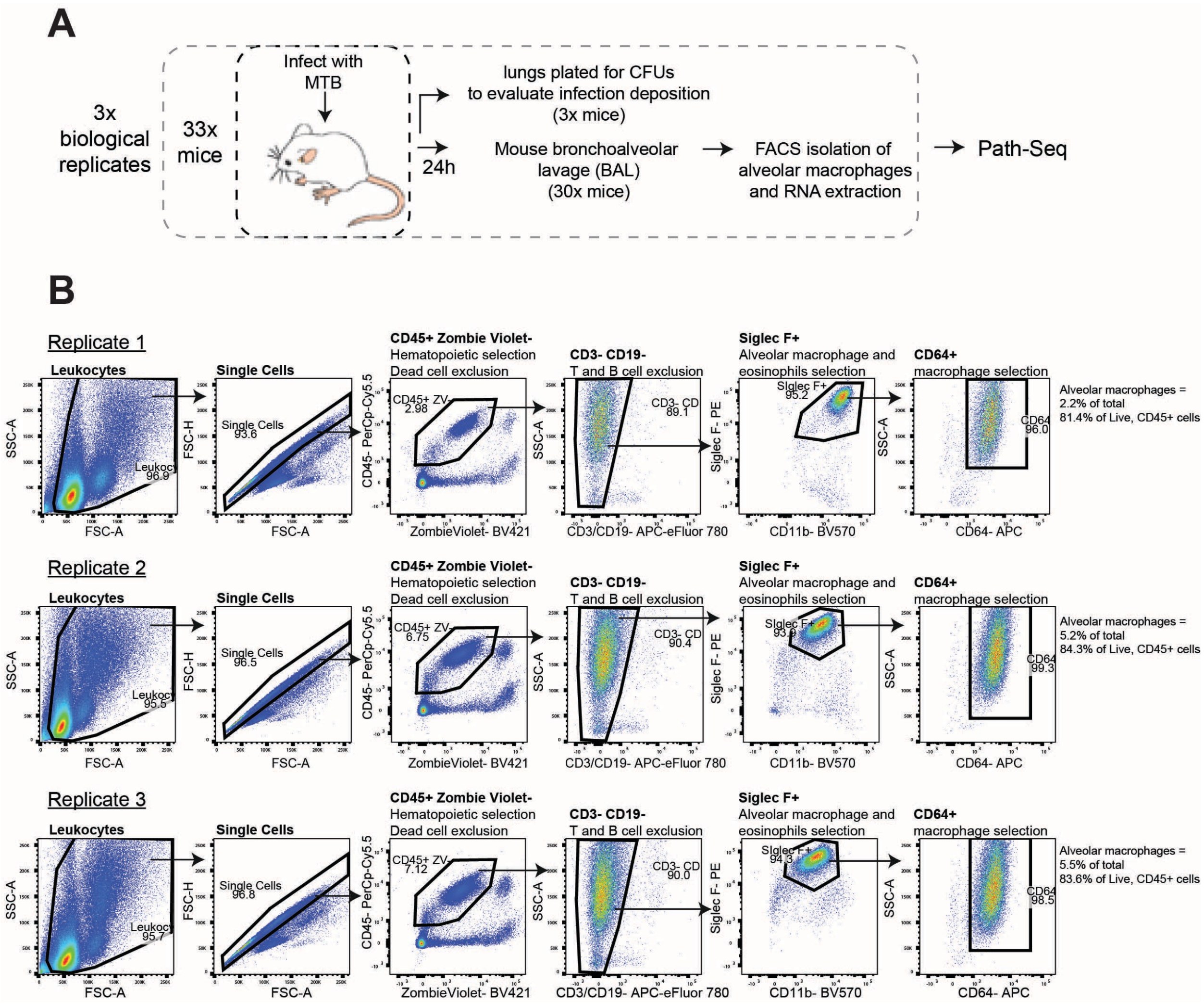

Fig. S1.
Schematic of *in vivo* infection and isolation of alveolar macrophages from MTB infected mice. (**A**) Schematic of *in vivo* infection. Infection, FACS-sorting and RNA extraction was repeated with three independent mouse infections. At 24 h post infection, the lungs of three mice were collected and plated for colony forming units (CFUs) to evaluate deposition of each infection (deposition reported in **Appendix Table S1**). Bronchoalveolar lavage (BAL) was performed on the remaining 30 mice, followed by FACS isolation of alveolar macrophages from BAL and RNA extraction. Path-seq was performed on all three replicates. (**B**) Flow cytometry analysis to sort alveolar macrophages from BAL of wt mice 24 h after aerosol infection of $6x10^3$ MTB. Cell viability was assessed using Zombie Violet viability dye. Alveolar macrophages were defined as $CD45^+$, $CD3^-$, $CD19^-$, $SiglecF^+$, $CD11b^{mid}$ and $CD64^+$. Plots show the percentages of cells from each parent population for each of the three replicate infections.

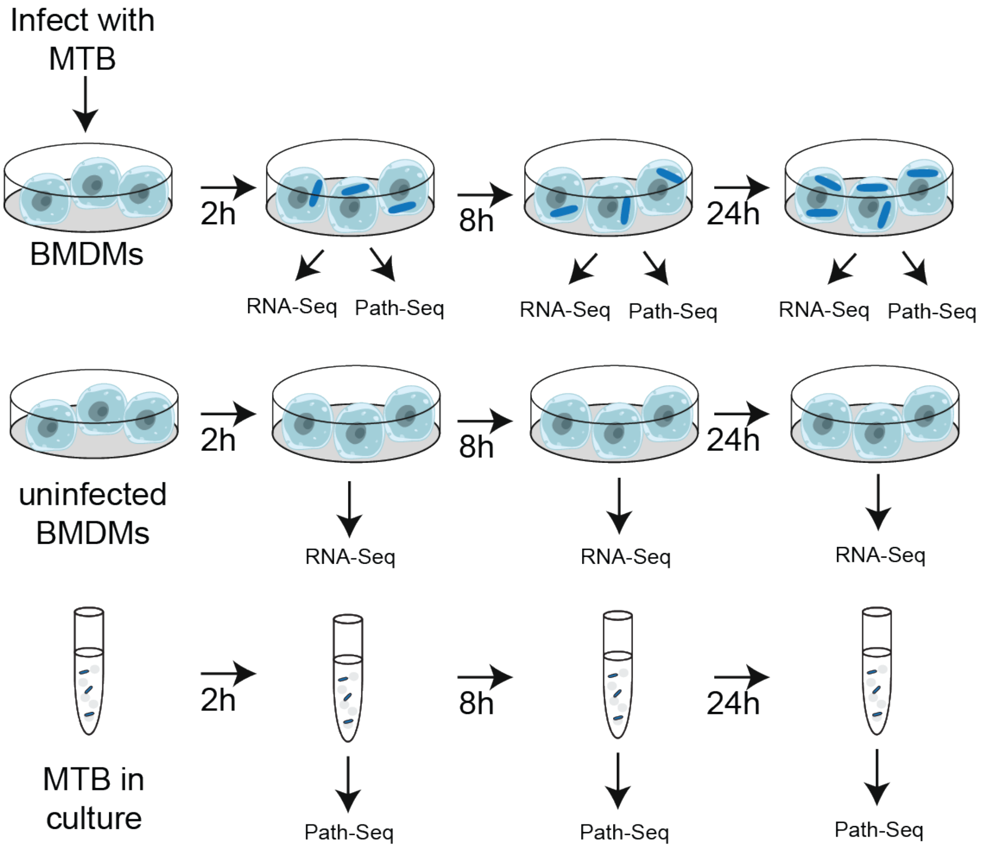

Fig. S2.

Schematic of *in vitro* infection. (**A**) Mouse bone marrow derived macrophages (BMDMs) were infected with MTB H37Rv at MOI of 10. MTB infected BMDMs were lysed with TRIzol at given time points and RNA samples were prepared for sequencing by RNA-seq and Path-seq (MTB enrichment), as described in main text. (**B**) Uninfected BMDMs were collected as a host control and processed by RNA-seq. (C) MTB grown in 7H9 broth culture were used as extracellular MTB control and processed by Path-seq.

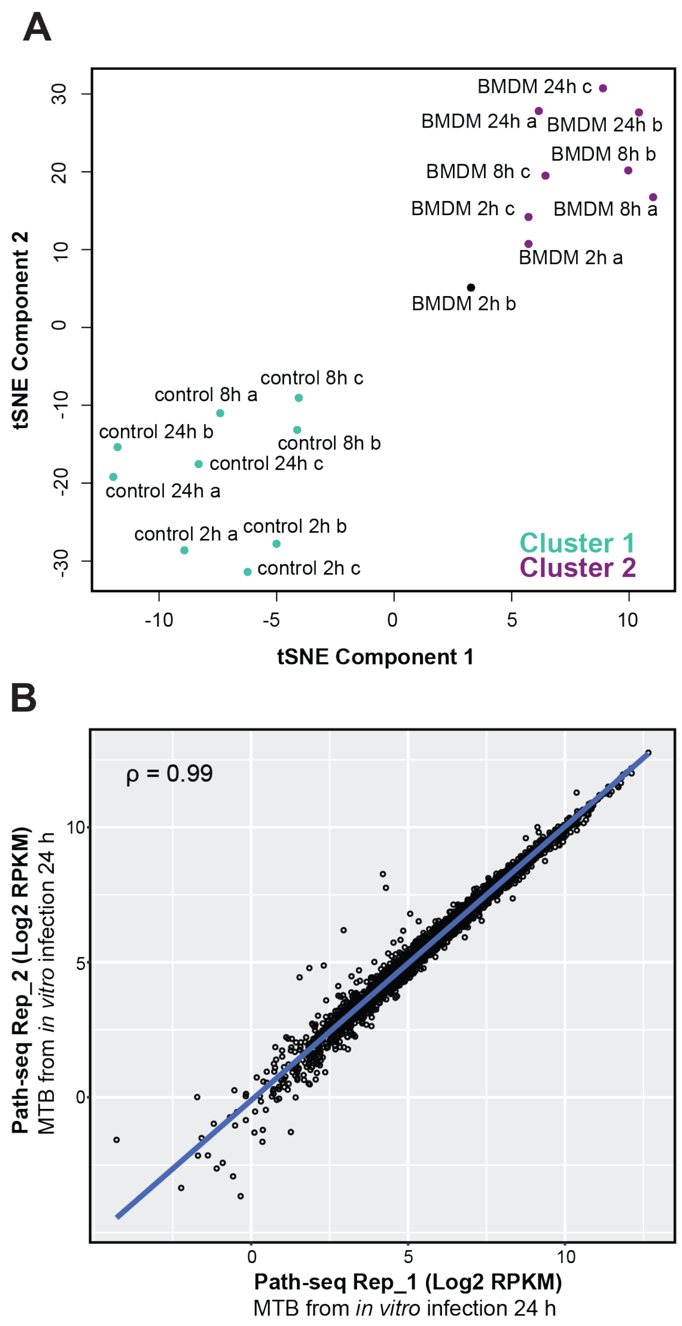

Fig. S3.

Analysis of biological Path-seq replicates from *in vitro* infection. (**A**) Two dimension t-SNE plot of Path-seq data from *in vitro* infection samples. MTB from infected bone marrow derived macrophages are labeled as “BMDM” and extracellular MTB grown in 7H9 are labeled as “control”. The t-SNE plot was constructed using the normalized read counts (produced by DESeq2) of 11354 genes with differential expression in one or more time points. Shown clusters were defined using the R function, NbClust (Charrad et al, 2014). (**B**) Correlation between replicates from *in vitro* infection collected at 24 h. Scatter plot of log2 RPKM values is shown with Pearson correlation, P-value < 0.0001.

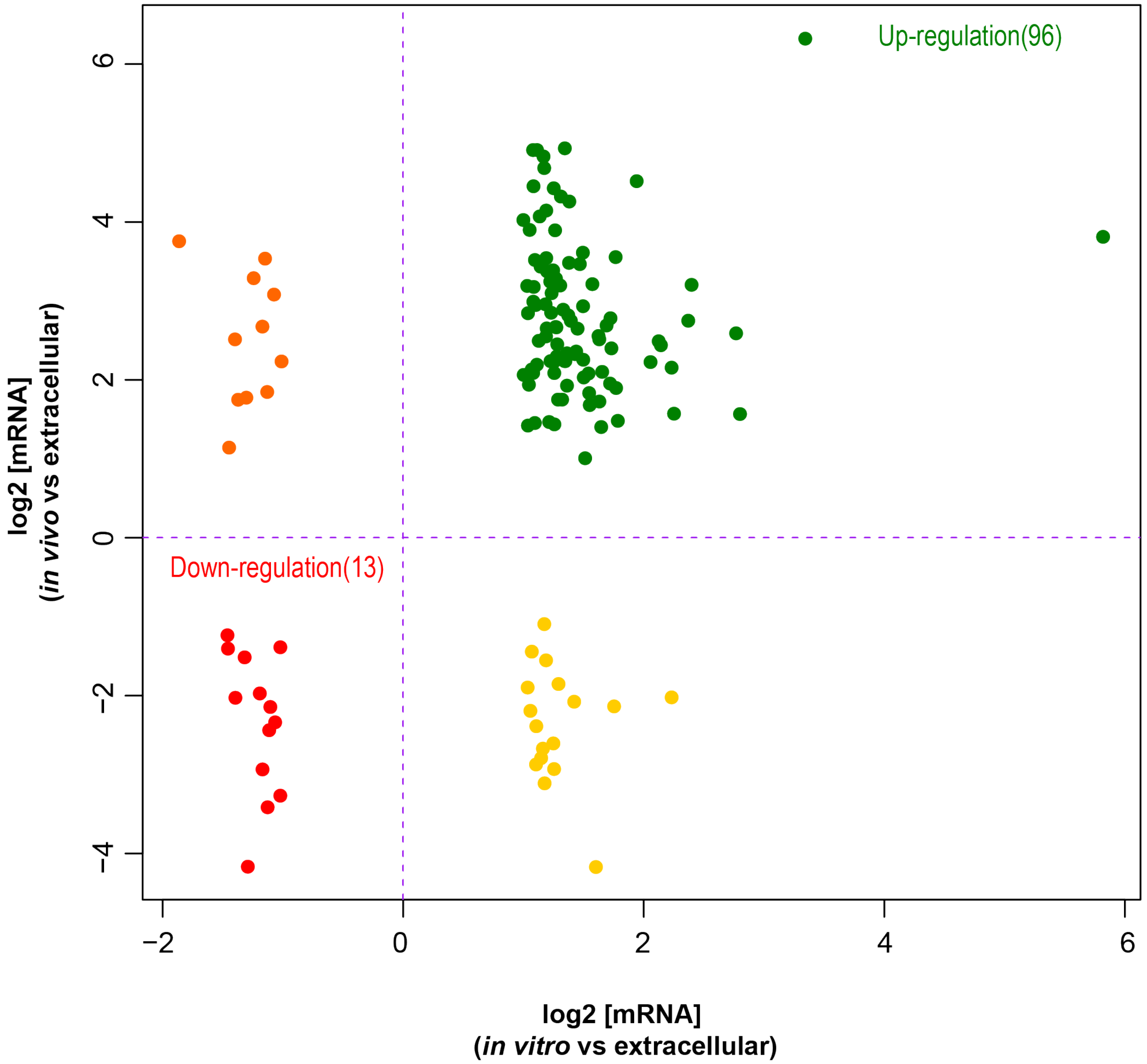

Fig. S4.

Analysis of significantly differentially expressed genes from both *in vitro* and *in vivo* infection models. Scatter plot of log2 fold change expression of MTB from infected BMDMs vs extracellular MTB and log2 fold change of MTB from alveolar macrophages of MTB infected mice vs extracellular MTB. This plot includes genes that were significantly differentially expressed at any time point. All samples were processed by Path-seq method.

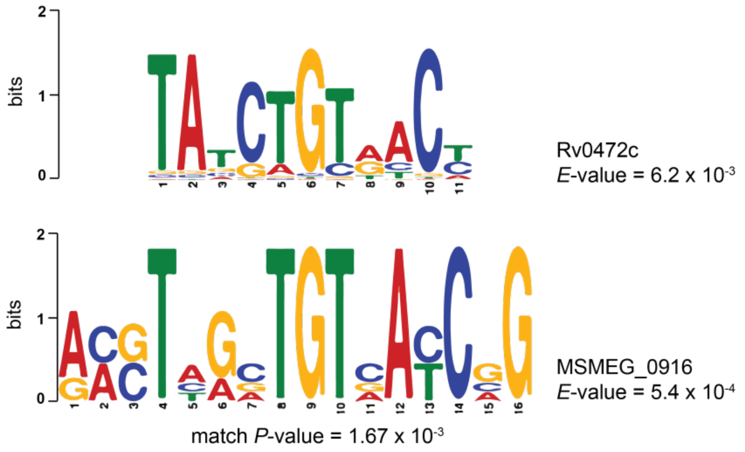

Fig. S5.

Consensus motifs from ChIP-seq peaks of *Rv0472c* overexpressed in MTB (top) and *MSMEG_0916* overexpressed in MSM (bottom). For consensus motif determination, we searched conserved DNA sequences within ±50 nucleotides of high quality (score > 0.7) ChIP-seq peak centers using MEME(Bailey & Elkan, 1994). Alignment of consensus motifs was performed with Tomtom(Gupta et al, 2007), match *P*-value = 1.67×10^{-3}.

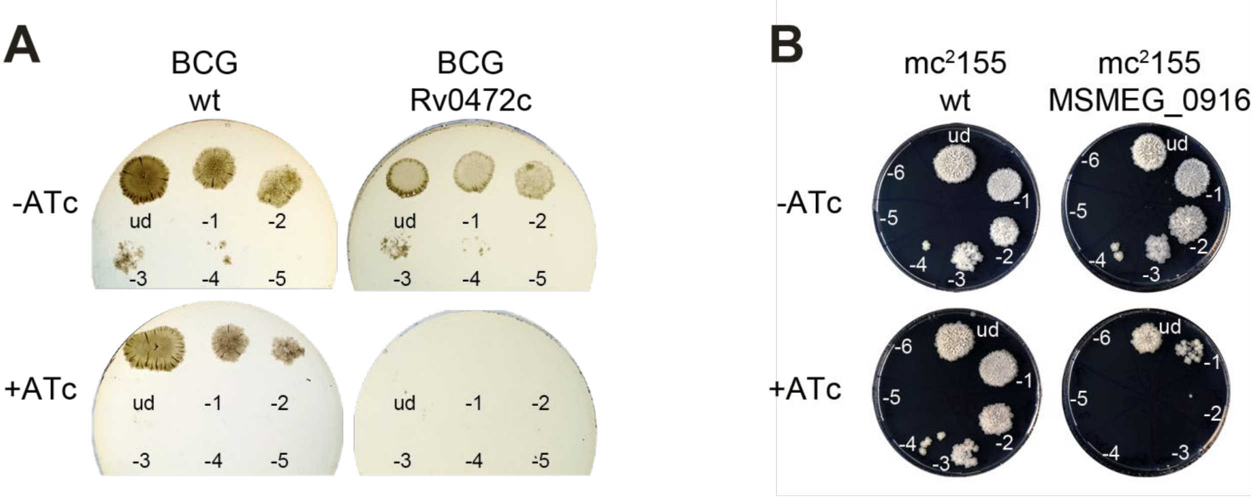

Fig. S6.
Cell viability of Rv0472c and MSMEG_0916, overexpression strains. (**A**) Cell viability of BCG overexpressing *Rv0472c*. Serial ten-fold dilutions of BCG wild type (wt) and BCG with inducible overexpression of *Rv0472c* were spotted on 7H10 agar plates with or without ATc. (**B**) Cell viability of MSM overexpressing MSMEG_0916. Serial ten-fold dilutions of MSM mc^{2}155 wild type (wt) and MSM with inducible overexpression of *MSMEg_0916* were spotted on 7H10 agar plates with or without ATc.

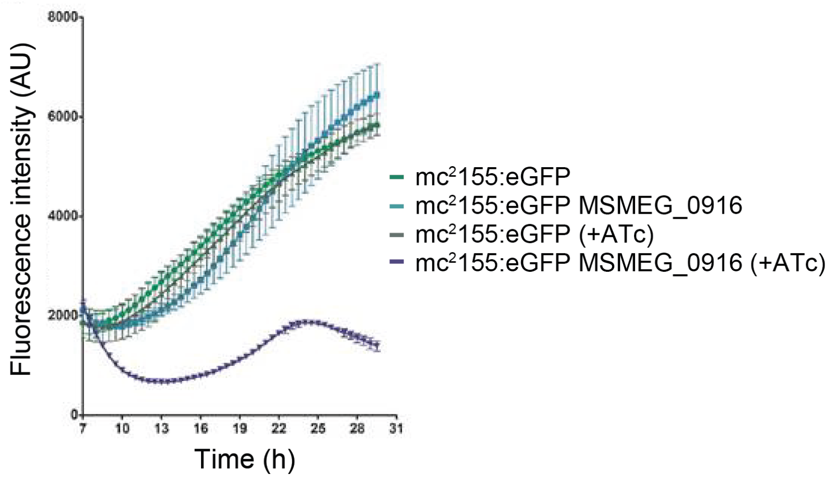

Fig. S7.

Growth of MSM overexpressing *MSMEG_0916* in 7H9 broth. MSM mc^{2}155 wt and mc^{2}155/pDTCF-MSMEG_0916 strains were transformed with an eGFP integrative vector. Growth of mc^{2}155 wt and mc^{2}155/pDTCF-MSMEG_0916 was monitored for fluorescence intenstiy (485/520 nm) in 96-well plates in the presence or absence of ATc.

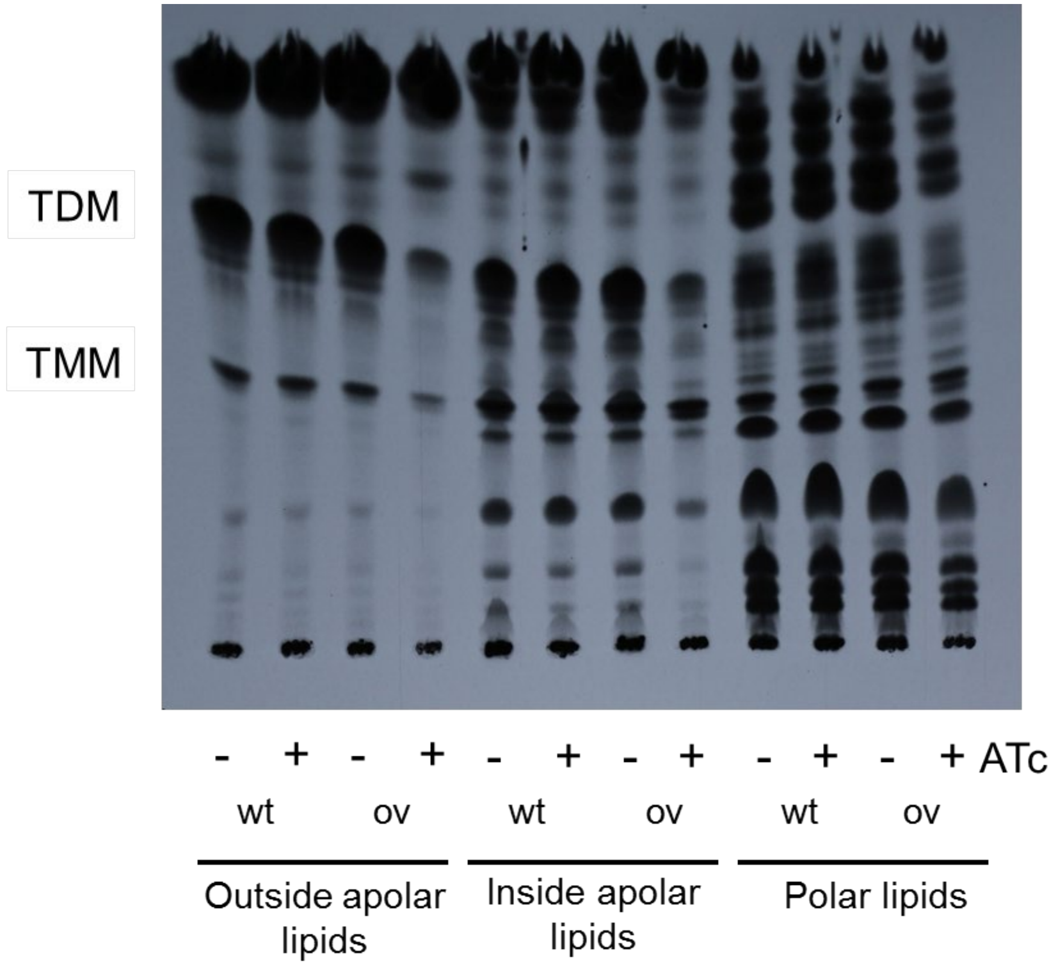

Fig. S8.
Assay of total mycolates in *MSMEG_0916* overexpression strain. TLC of outside, inside and apolar lipids extracted from MSM wildtype (wt) and MSM overexpressing *MSMEG_0916* (ov) in the presence (+) or absence (-) of ATc. Trehalose dimycolate (TDM) and trehalose monomycolate (TMM) were extracted following labeling and analysed by autoradiography-TLC using equal counts (15,000 cpm) for each lane.

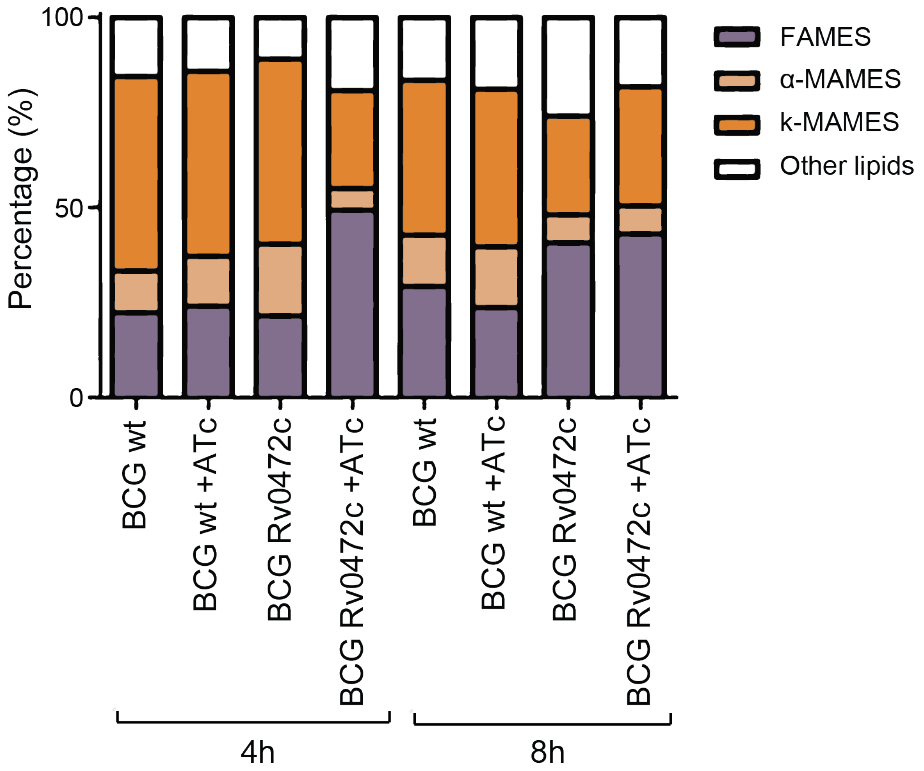

Fig. S9.

Densitometric analysis of FAMES and MAMES in BCG *Rv0472c* overexpression strain. Bar graph showing the relative amounts of ^{14}C-labeled methyl esters from BCG wildtype (wt) and BCG overexpressing *Rv0472c* species with or without ATc for 4 h and 8 h. The methyl ester amounts are indicated as percentages of total amounts of ^{14}C-labeled methyl esters detected on the TLC plate shown in **Fig. 5C**, as determined by densitometry. FAMES; fatty acyl methyl esters. α-MAMES; α-mycolic acid methyl esters. k-MAMES; keto-mycolic acid methyl esters.

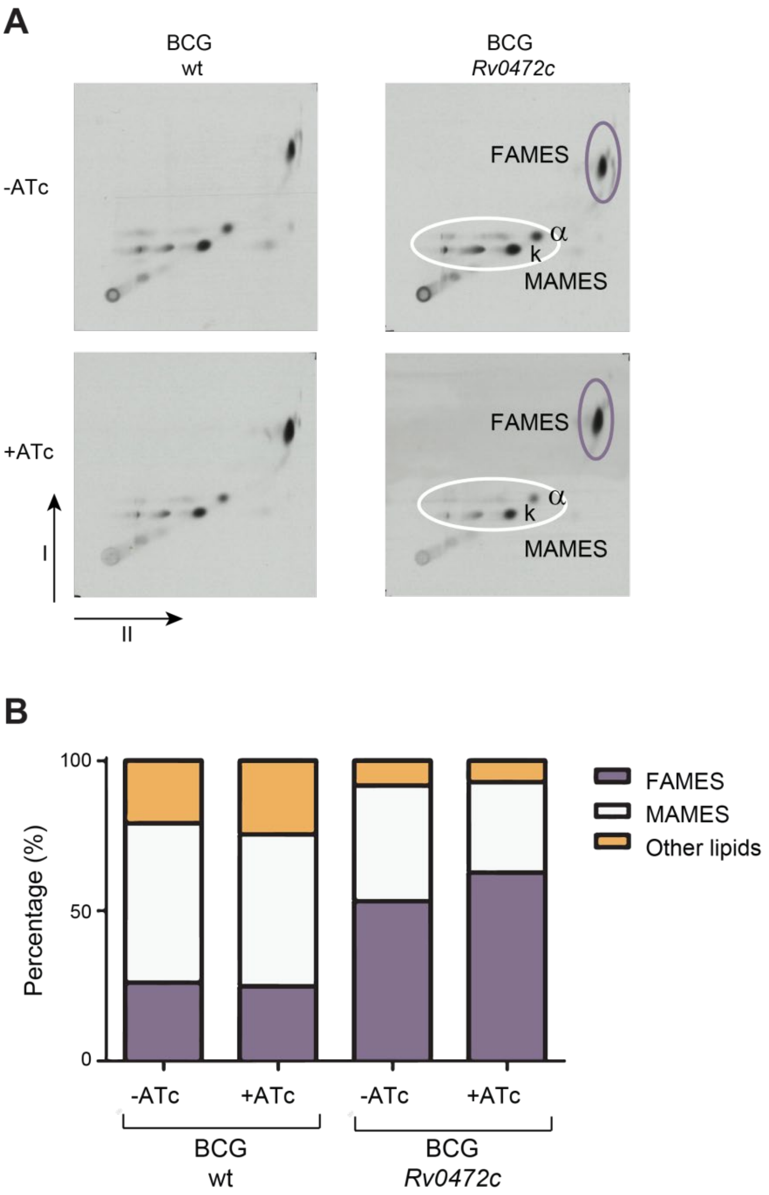

Fig. S10.
A 2-D TLC analysis of FAMEs and MAMEs from BCG overexpressing *Rv0472*c. (**A**) 2D-argentation TLC analysis of fatty acid methyl esters (FAMEs) and mycolic acid methyl esters (MAMEs) from inside apolar lipid extracts of BCG wildtype (wt) and BCG overexpressing Rv0472c in the presence or absence of ATc. Alpha(α)-MAME and keto(k)-MAME species are indicated. (**B**) Bar graph showing the relative amounts of ^{14}C-labeled MAMES and FAMES as percentages of total amounts of ^{14}C-labeled methyl esters detected on the 2D TLC plates shown in **A**, as determined by densitometry.

	Non-zero genes (%)	Total read counts	Mean count*	Coefficient of variation*
AM T24h (deposition = 8100 CFUs)	49.23	997862.9	359.07	1.93
AM T24h (deposition = 5633 CFUs)	31.12	1077297.3	613.14	2.03
AM T24h (deposition = 4333 CFUs)	14.28	183383.2	227.52	1.80
extracellular 24h	96.88	1588365.6	290.42	2.00
extracellular 24h	97.47	2867568.6	521.18	1.95
extracellular 24h	96.63	1603930.7	294.02	1.97

Table S1.

Summary analysis from Path-seq data of alveolar macrophages (AMs) from MTB infected mice and extracellular MTB grown in 7H9 broth. Non-zero genes were counted as any gene with one or more reads. The total read counts per sample were determined based on reads that aligned to MTB genome. Mean count was calculated as the mean read count across all non-zero genes in the samples. Coefficient of variation was calculated as standard deviation divided by the mean across all non-zero genes in the samples. Asterisk (*) indicates only non-zero reads were used in the analyses.

	Rohde et al, 2007	This study	Overlap	*P*-value
Total Features	4322	5622	4160	NA
DEGs 2h	68 (62)	746 (539)	29	5.77 E-11
	123 (118)		60	5.49 E-24
DEGs 24h	186 (177)	412 (277)	41	3.17 E-13

	Schnappinger et al, 2003	This study	Overlap	*P*-value
Total Features	3776	5622	3726	NA
DEGs 24h	599 (586)	412 (233)	100	6.6 E-25

Table S2.

Comparison of significantly differentially expressed genes (DEGs) from in vitro Path-seq data with published microarray-collected transcriptomics data of MTB-infected macrophages (Data Ref: Rohde et al, 2007, Rohde et al, 2007, Data Ref: Schnappinger et al, 2003, Schnappinger et al, 2003). Top table: comparison with Data Ref: Rohde et al, 2007. Bottom table: comparison with Data Ref: Schnappinger et al, 2003. Total features from this study (5622) included non-coding RNA sequences, UTRs, etc. The numbers in parentheses indicated the number of genes present in both datasets and the values used for hyper-geometric test. The *P*-values represent the calculated significance associated with the hyper-geometric test. The comparison with Rohde et al, 2007 shows two comparison at 2 h, based on different tools and thresholds the authors used to determine significantly expressed genes in their study. In the comparison with Schnappinger et al, 2003, we used the set of DEGs defined by the authors using absolute fold-change > 1.5 and *P*-value < 0.05 thresholds.

Dataset EV1. (separate file)

Significantly differentially expressed genes determined using DESeq2 of MTB from *in vivo* infection (isolated from alveolar macrophages from MTB infected mice at 24 h post infection) vs extracellular MTB (cultured in 7H9 broth for 24 h) using Path-seq. Columns D-H are the rounded raw read counts from DuffyNGS.

Dataset EV2. (separate file)

Significantly differentially expressed genes determined using DESeq2 of MTB from in vivo infection (isolated from bone marrow derived macrophages from MTB infected mice at 2, 8, and 24 h post infection) vs extracellular MTB (cultured in 7H9 broth for 2, 8, and 24 h) using Path-seq. Columns D-I of each worksheet are the rounded raw read counts from DuffyNGS.

Dataset EV3. (separate file)

Dataset contains comparisons of significantly differentially expressed genes between the in vivo and in vitro infections.
